# Alternative career pathway decision-support job database for international medical graduates in Canada

**DOI:** 10.1186/s13104-022-06232-8

**Published:** 2022-10-29

**Authors:** Saif Sikdar, Nashit Chowdhury, Deidre Lake, Tanvir C. Turin

**Affiliations:** 1 Department of Microbiology, Immunology and Infectious Disease, Cumming School of Medicine, University of Calgary, Calgary, AB Canada; 2 Alberta International Medical Graduates Association, Calgary, AB Canada; 3 Department of Family Medicine, Cumming School of Medicine, University of Calgary, Calgary, AB Canada; 4 Department of Community Health Sciences, Cumming School of Medicine, University of Calgary, Calgary, AB Canada

**Keywords:** International medical graduates, Internationally trained health professionals, Alternative careers, Jobs, Professional integration, Career support tool, Career decision

## Abstract

**Objectives:**

Canadian regulations have made it challenging for the international medical graduates (IMGs) to get jobs in their original profession as physicians. Consequently, alternative careers are gaining interest among IMGs to avoid underemployment or unemployment. We conducted research to identify the factors that IMGs consider for taking up an alternative career in Canada. Based on those understandings, we aimed to create a database where information about health-related alternative jobs is presented in a searchable way, which can aid IMGs’ strategic job search.

**Data description:**

We first determined job searching preferences and constraints for IMGs regarding alternative career through focus groups. We used their preferred and constraining factors for collecting job-specific information through systematically reviewing job advertisements. Using this information, we created a database that contains available alternative career pathways for IMGs living in Canada. In total, we have identified 1374 job titles under 192 unique job categories comprising 47 National Occupational Classification (NOC) codes that could be suitable for IMGs seeking an alternative career based on their own short, intermediate, and long-term career goals. We expect that this database will help IMGs in deciding on alternative careers.

## Objective

Canada accepts thousands of skilled immigrants each year to fulfill the workforce need of various sectors [1]. These immigrants leave their home country with a hope of making a better life for themselves and their families through progressing in their field of expertise, however, a successful job integration can be challenging for some professional groups such as the international medical graduates (IMGs) [2–4]. Under current rules and regulations, IMGs living in Canada are defined as those who have received their academic and professional training outside of Canada or the USA. After clearing all pre-requisite exams, they need to compete for residency training seats under a restrictive quota system to get licensure to practice as physicians in Canada [5–9]. Unsuccessful IMGs usually keeps re-applying and join the new applicant pool. This leads to an ever-increasing number of applicants causing a bottleneck situation as the spots for residency program are not accommodative for the increasing number of applicants.

Alternative career pathways (ACPs) in health and wellness sector could be a significant viable option towards mitigating the employment problem of IMGs [10], which needs to be strategically planned. For this professional group, a suitable alternative career could be a job where they can use their health-related knowledge and technical skills. However, there is a lack of understanding, information, and organizational support on ACPs for IMGs [8–10]. Our program of research is built around the objective to identify and facilitate suitable alternative career pathways available for IMGs in Canada. We conducted a study to identify the factors that IMGs considered when they explored an alternative career. Using that learning, we have developed a database with important job-specific information that will serve as a career decision-making tool for the IMGs in Canada [4].

## Data description

### Data collection and database development

We engaged with IMGs to identify the factors they consider when searching for an alternative career through Alberta International Medical Graduate Association. Through focus groups with IMGs, we have identified number of factors those are considered by them when they explore ACPs in Canada. These factors incudes; if the job is a clinical or non-clinical type job, whether the job is regulated or non-regulated, which domain of health and wellness the job belongs to, what activities are associated with job, educational requirements needed for the job, working environment associated for the job (indoor or outdoor location, hazards associated, etc.), minimum and maximum salary for the job, experience requirements, usefulness of existing transferable skills of different kinds, flexibility of the work, job demand, If any bridging opportunities are available or not, length of additional training required for the job, availability of flexible training options, availability of practicum/internship/co-op opportunities, future growth or career progression opportunities, etc. Figure 1 (data file 1 in Table 1) summarizes the steps followed for creating an ACP database for IMGs.

**Table 1:** Overview of data files/ data set

Label	Name of data file/data set	File types (file extension)	Data repository and identifier (DOI or accession number)
Data file 1	Figure 1: Steps followed for creating an ACP database for IMGs	PDF file (.pdf)	Figshare (https://doi.org/10.6084/m9.figshare.20138342) [11]
Data file 2	Figure 2: ACP database contains job-specific information collected from different sources	PDF file (.pdf)	Figshare (https://doi.org/10.6084/m9.figshare.20138336) [12]
Data file 3	Table: Description of the variables included in the alternative career pathway decision-support job database for international medical graduates in Canada	PDF file (.pdf)	Figshare (https://doi.org/10.6084/m9.figshare.20522265) [13]
Data file 4	Dataset: ACP decision-support database for IMGs in Canada	MS Excel file (.xlsx)	Figshare (https://doi.org/10.6084/m9.figshare.20138339) [14]

Next, based on the identified aspects, collected job-specific information (27 variables) through systematically reviewing job advertisements, government, and other institutional job service websites. We collected data from several sources such as job advertisements, government websites, and existing career-specific databases. These sources included—(1) National Occupational Classification (NOC) system, which is Canada’s national system for describing occupations, (2) the Career Handbook created by Employment and Social Development Canada and Statistics Canada; (3) job advertisements from Indeed, Google, and other job search sites; (4) job bank and government career services; (5) educational institutions websites; and (6) hospitals’ and clinics’ career websites. Finally, we compiled the data and used unique codes for the values of the variables where necessary to develop an accessible searchable database. Figure 2 (data file 2 in Table 1) shows the major sources of the job-specific information used for creating this ACP database. Description of the 27 variables included in the alternative career pathway decision-support job database are provided in a Table (data file 3 in Table 1).

### Key features of the database

The ACP Job dataset (data file 4 in Table 1) contains 1,374 job titles under 47 NOC codes that are suitable for IMGs seeking to explore alternative health and wellness sector careers in Canada based on their short-, intermediate- and long-term goals. After critical review and job description analysis according to the IMGs’ preferences, we have identified 192 unique job categories. There are primarily two categories of jobs: clinical (35.42%) and non-clinical (64.58%). Interestingly, we have found that around 7.35% of clinical and 29.84% of non-clinical job categories do not require any sort of license or approval from any regulatory bodies. Although most other jobs require a certain level of training, certificate, or license from the respective licensing authorities, obtaining those regulatory approvals appears to be easier compared to the license to practice as a physician.

In conclusion, the ACP database we have compiled contains information covering the points which IMGs generally consider regarding an alternative career in Canada. We expect that this database will help IMGs in the search and decision making on alternative careers choices suitable for them and help them to strategize for these jobs. This database will also be helpful for resettlement support providing organizations and career counsellors helping IMGs to develop effective programs, counselling and guidance materials to support IMGs’ for job market integration.

## Limitations

The data collection was conducted from 01 June 2021 to 30 November 2021. Data collected from job advertisements limited within this period. Thus, it is possible that later job advertisements from different companies require somewhat more or less job-related experience and qualifications. However, multiple job advertisements were used to collect information that may limit this issue.

## Data Availability

The data described in this Data Note manuscript can be freely and openly accessed on Figshare under DOI: Fig. 1 https://doi.org/10.6084/m9.figshare.20138342 [11], Fig. 2 https://doi.org/10.6084/m9.figshare.20138336 [12], Table: https://doi.org/10.6084/m9.figshare.20522265 [13], and Dataset: https://doi.org/10.6084/m9.figshare.20138339 [14]. Please see Table 1 for details.

## Abbreviations

IMGs: International Medical Graduates
NOC: National Occupation Classification
ACP: Alternative Career Pathway
